# Impact of early dam contact on veal calf welfare

**DOI:** 10.1038/s41598-022-25804-z

**Published:** 2022-12-22

**Authors:** L. E. Webb, F. Marcato, E. A. M. Bokkers, C. M. Verwer, M. Wolthuis-Fillerup, F. A. Hoorweg, H. van den Brand, M. B. Jensen, C. G. van Reenen

**Affiliations:** 1 Animal Production Systems Group, Wageningen University & Research, Wageningen, The Netherlands; 2 Adaptation Physiology Group, Wageningen University & Research, Wageningen, The Netherlands; 3 Louis Bolk Institute, Bunnik, The Netherlands; 4 Animal Health & Welfare Group, Wageningen Livestock Research, Wageningen University & Research, Wageningen, The Netherlands; 5 Behaviour Stress and Welfare Group, Aarhus University, Tjele, Denmark

**Keywords:** Animal behaviour, Animal physiology

## Abstract

Dairy calves, including surplus calves, are typically separated from their dam within hours of birth. The aim of this study was to assess the welfare impacts of raising surplus calves destined for veal with their dam for 2 or 4 weeks until transport. Surplus calves from one dairy farm were separated from their dam at birth (n = 39) or kept with the dam (n = 37) until transport to the veal farm at either 2 (n = 50) or 4 (n = 26) weeks of age, with abrupt separation for dam-reared calves. Calf measures of body weight, health, immunity, haematology and behaviour were recorded at the dairy and veal farms. Dam-reared calves had higher body weights in weeks 3, 4 and 5 at the DF, as well as at arrival at the veal farm, but by slaughter this advantage was lost. More dam-reared calves had fever in week 3 and showed signs of disease in week 5 at the dairy farm. Dam-reared calves did not differ in IgG, IgA or IgM levels but had higher counts of white blood cells, which could reflect a higher pathogen exposure rather than improved immunity. Dam-reared calves displayed more fear towards humans in a human approach test at 5 and 7 weeks after arrival at the veal farm, and more frequent social behaviours at the veal farm at 9 and 16 weeks of age. In conclusion, it seems that there may be both advantages and disadvantages to keeping veal calves with the dam in terms of welfare in the current system.

## Introduction

It is common practice for dairy farmers to separate the calf from its dam within hours of birth^1^. The main reasons for this practice are protecting the calf against diseases (e.g. Johne’s disease, also called paratuberculosis), monitoring the health and colostrum/milk intake of the calf, ensuring the calf gets socialised with human handlers, separating mother and young before a strong bond is formed, preventing the calf from drinking saleable milk, and ensuring adequate milk let-down in the milking parlour^2–4^. This practice has, however, been criticised by the public who views it as unnatural^5–7^. Informing the public about the reasons for the separation of calf and dam appeared not to change these public concerns^6^. Furthermore, it has been linked to concerns regarding the welfare of cow and calf. Animal welfare is defined here as the balance between positive/pleasant and negative/unpleasant experiences, where a good life is defined as one where the positives significantly outweigh the negatives^8,9^.

Animal welfare concerns linked to early dam-calf separation include: (1) the thwarting of natural behaviours and positive experiences linked to affiliative behaviours between mother and young, e.g. nursing and bonding^3,10–13^ and subsequent higher prevalence of abnormal behaviours (e.g. cross sucking)^14^, (2) increases in certain diseases, such as calf diarrhoea^15^ and dam mastitis^16,17^, and (3) lower weight gain of the calf^15^. Early dam-calf separation has moreover been linked to poorer social skills and reduced sociability (as a personality trait) in the calves^18–21^ and later when they become adult^22,23^. Compared to dam-reared calves, calves separated shortly after birth show lower levels of specific social behaviours, including social play, submissive behaviours and agonistic behaviours^14^. Improved social skills and sociability could promote future welfare via an increase in positive social interactions and decrease in negative social interactions^24,25^, although this may also be promoted by social contact with other calves^14^.

An alternative to early cow-calf separation is to keep the dam and the calf together for a period of time. In comparison with separation at birth, dam-rearing could hypothetically lead to an increase in colostrum intake, as calves have the opportunity to suckle, possibly in addition to being hand-fed colostrum by the farmer^26^. A higher colostrum intake in the first hours following birth is linked to improved transfer of passive immunity, increased serum immunoglobulin G (IgG), improved health and a better body weight gain^27,28^. Contact with the dam, regardless of colostrum and milk intake, has also been linked to better IgG absorption in the calves^29^. Further, calves nursed by their dam are likely to have access to more milk across more meals which may further increase body weight gain^18^.

Most of the research conducted on the impacts of dam-rearing on calf welfare, however, was conducted on the heifer calves that stay on the dairy farm for herd replacement. To the authors’ knowledge, no such research has ever been conducted on the surplus calves that leave the dairy farm to be fattened for veal. Given the beneficial effects of keeping calves longer with their mothers on calf health, behaviour and performance, it is relevant to investigate this topic in calves destined to veal production as dam-rearing might have positive effects on the robustness of calves at the veal farm. Increasing the robustness of calves destined for veal would mitigate disease and antibiotic use, thereby improving animal welfare and economic performance of the veal industry, and hence contribute to improving the overall sustainability of veal products^30^. Robustness is the capacity to maintain a given state in the face of challenge^30^, and is linked to a lower risk of morbidity and mortality. Past research has found links between indicators of robustness and lower future risk of morbidity or mortality, including higher body weight, higher IgG level, higher lymphocyte count, lower neutrophil count, being male and having Belgian blue genes^31–36^. It is hence highly relevant to investigate whether dam-rearing on dairy farms affects these indicators of calf robustness on the veal farm.

Despite the numerous advantages of dam-rearing, one disadvantage of this practice is a decrease in machine milk yield of the dam, which may or may not reach normal levels once the calf is removed^4,17,37^. Another disadvantage is that the short period in which veal calves remain on the dairy farm makes it difficult to apply gradual (2-step) separation from the cow, which has been found to reduce the stress of separation for both cow and calf^38,39^. This higher stress level may lead to poorer performance at the veal farm, as separation stress combined with transport stress have been linked to an increased susceptibility to disease^40,41^. Veal calves are usually transported from the dairy farm to a veal farm from 2 weeks of age. At this age, there is an immune gap between the passive transfer of maternal immunity and the development of the calve’s own immunity, further increasing its susceptibility to disease^42^. Finally, dam-reared calves are likely to be more fearful of humans^21^, although this may be less of an issue in veal calves compared with dairy calves because they are not often handled and are slaughtered at 6 months. There is therefore a need for more insight into the effects of dam-rearing on the welfare of veal calves to understand whether this practice is effectively beneficial for these animals.

The main aim of the present study was to assess the impact of dam-rearing on the welfare of veal calves and the productivity of their dams. Moreover, since age at first transport to the veal farm can have an important impact on calf robustness, the interaction between transport age (2 weeks vs 4 weeks) and dam-rearing was also investigated. The current study focused on indicators of calf welfare and robustness, including body weight, immunoglobulins, haematological profile, fear of humans and social behaviour. Moreover, to assess productivity of the cows, the dry period length, lactation length and machine milk yield were recorded. The expectations were as follows:

1. Dam-reared calves show improved immunity, weight gain, health at the dairy and veal farm, display more social behaviours towards pen mates and display higher fear of humans at the veal farm.
2. Calves transported at 4 weeks, instead of 2 weeks, show higher weight gain, and improved immunity and health at the veal farm. No impact on social behaviour or fear of humans is expected.
3. Machine milk yield is lower during the time that cows are nursing their calves, but rapidly restores following removal of the calf, while dry period length and lactation length remain unaffected.

## Materials and methods

The experiment and all associated protocols were approved by the Central Committee on Animal Experiments (the Hague, the Netherlands; approval number 2017.D-0029) and carried out in accordance with relevant guidelines and regulations. Permission to study the animals was obtained by the dairy farmer and veal farmers. The methods are reported following ARRIVE guidelines. The experiment ran from March 2019 to May 2020, on one Dutch dairy farm and eight Dutch veal farms. This study was part of another larger study which investigated the effect of transport age on calf robustness, hence these methods are also described in sister manuscripts^43,44^. This means that we had no control over the sample size in the present study.

### Animals and treatments

This study was a 2 × 2 factorial design, with rearing practice (with or without dam during the entire time on the dairy farm) and transport age to the veal farm (2 or 4 weeks) as factors. The experimental unit was the animal. The treatment of transport age was allocated based on the week in which a calf was born. Calves born in the first two weeks from the start of the experiment left the dairy farm at 4 weeks of age, while calves born in the following two weeks left the dairy farm at 2 weeks of age. Calves born within this 4 weeks timeframe were transported to the same veal farm: calves born in week 1 and 3 were transported to the veal farm in week 5, while calves born in weeks 2 and 4 were transported in week 6, to the same farm, meaning that per veal farm, 2 transport moments were performed. At each transport day, one transporter (the same throughout the entire study) collected the calves and brought these directly to the veal farm. This 4 weeks timeframe was repeated throughout the experiment 8 times (to 8 veal farms). The exact age at transport was (mean ± SEM [range]): 2 weeks transport = 19.5 ± 0.3 (16–22) days; 4 weeks transport = 33.1 ± 0.4 (30–36) days. The body condition score of the dams was noted at parturition by the dairy farmer, using a scoring system from 1 (severely underweight) to 5 (severely over weight), based on a scheme found at Ketolution.com (Elanco).

#### At the dairy farm

**Figure 1:**
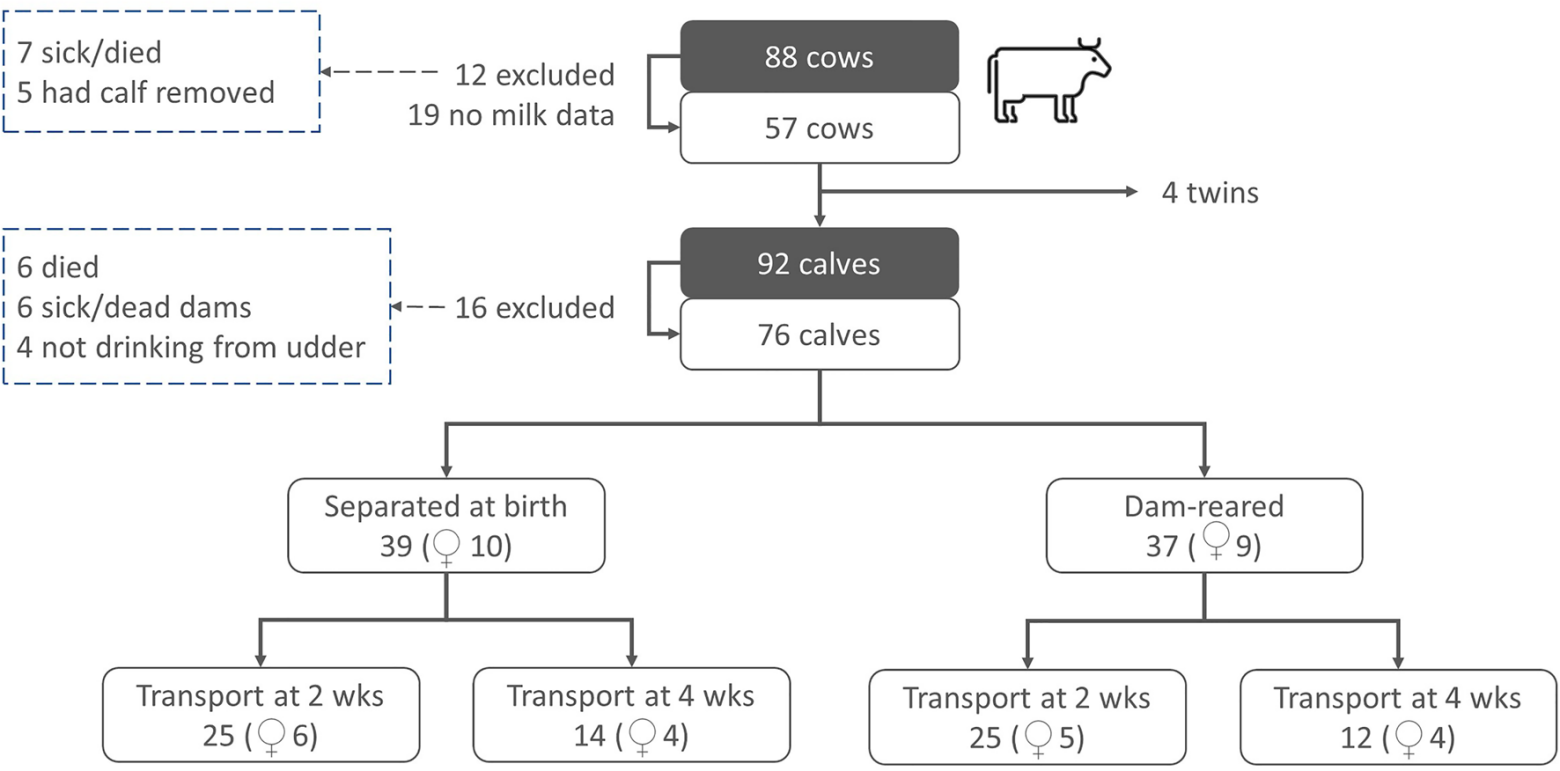
Animal numbers.

The dairy cows were loose-housed in deep compost housing for 200 cows with four Lely Astronaut A4 milking robots (Lely Industries, Maassluis, the Netherlands). The cows had free and voluntary access to pasture between April and November, following the first morning milking. Cows which produced calves for herd replacement were inseminated with semen from a Holstein Friesian (HF) bull, while other cows were inseminated with semen from a Belgian Blue (BB) bull for meat traits. In the first case, only the male calves were included in this study, while in the latter case, both males and females were included in this study. See Fig. 1 for animal numbers used in this study and note that there were 23 HF bull calves (8 dam-reared), 34 BB bull calves (20 dam-reared) and 19 BB heifer calves (9 dam-reared) ultimately used in the study. We hence had more BB calves than HF calves. Most calves were fed colostrum by the farmer (mean ± SEM): separated at birth = 11.7 ± 1.1 L; dam-reared = 3.0 ± 0.5L (in total). The average number of colostrum feedings by the farmer was: separated at birth = 3.0 ± 0.3; dam-reared = 0.8 ± 0.1 feedings. Of note: three dam-reared calves received colostrum from a cow other than their own mother; 14 calves did not receive colostrum from the farmer and were left to drink from their mother, under supervision (12 dam-reared and 2 separated at birth).

If the calves were separated from their dams at birth, they were moved to an igloo (300 × 70 cm) following colostrum feeding, within 12 h of birth. These calves were not assisted in drinking from their dam’s udder. The igloos were outdoor individual shelters with a small run (approximately 70 × 150 cm) and straw bedding inside and outside the igloo. Separated calves received warm whole milk from the bulk tank twice per day in teat buckets attached to the front of the pen: week 1 = 2 × 4 L, week 2 = 2 × 4.5 L, week 3 = 2 × 5 L and week 4 = 2 × 5.5 L. They received additional concentrate in a trough, although this was inconsistent, i.e. concentrate feed was not always present. These calves remained in these igloos for 2 or 4 weeks until transport to the veal farm.

The calves that stayed with their dams needed to learn to drink milk from the udder. The cows calved in a large calving/dry pen (9 × 30 m) with other peri-parturition and dry cows. This led to difficulties in calves finding their dam’s udder, in which case the farmer assisted the calves in suckling from their dam’s udder. The dam-reared calves and their dam stayed approximately 3 days in the calving pen. These calves remained with their dams on a fulltime basis – they could also follow their dam into the milking robot. When the cows went out to pasture, however, the calves, who were free to follow, typically did not follow their dam and remained in the deep-bedded compost barn (personal observation of the farmer). On the day of transport to the veal farm, these calves were abruptly separated from their dams and transported to the veal farm directly. This study originally included 92 calves and 88 cows (4 twins), of which we could use 76 calves and 57 cows, see Fig. 1.

#### At the veal farm

Eight veal farms were included in this study and each veal farmer used personal management decisions which were not influenced by the study. All eight veal farms were part of the same Dutch veal integration, which means they fed the same diet to their calves and followed similar veterinarian protocols. Our experimental calves were grouped with un-experimental calves and treated in accordance with the standard procedure at the veal farm. Veal farmers were blind to the rearing treatment of the calves. However, calves transported at 2 weeks wore different coloured eartags to calves transported at 4 weeks. At arrival, calves were first housed in individual pens within the larger pens, which allowed visual and auditory contact with other calves, and tactile contact with calves in adjacent pens. The calves typically remained in these individual pens for 3 weeks and were thereafter released into the group pens, of 4 to 10 calves, depending on the veal farm. Calves were fed milk replacer in buckets or troughs, typically without teats, and solid feed in troughs. The solid feed mainly consisted of concentrate (approx. 85%) with some chopped straw. The calves received both antibiotic and other medicine treatments, including anti-inflammatory and anti-coccidial treatments, and multivitamins when necessary as individual treatments. These individual treatments were recorded (see measurements section below). Moreover, calves received an average of 4.4 group treatments with antibiotics and an average of 3.9 group treatments with medicines other than antibiotics at the veal farm (these are typically given for outbreaks of bovine respiratory disease). These antibiotic treatments were provided via the milk for an average of 10 feedings over 5 days per treatment. Haemoglobin was measured twice during the fattening period by blood sampling of the jugular vein and calves that had low haemoglobin (< 5.5 mmol Hb) received extra iron (sub-cutaneous injection).

### Measurements

At the dairy farm, calves with their dams were captured one day a week (at approximately the same time each week and on the same day) and placed into an outside group pen together. At this time, all calves from all treatments were weighed and scored for clinical health on a weekly basis until transport to the veal farm. Calves born within the past week relative to the visit and calves due to be transported the next day were also blood sampled. The blood sampling was always performed by one of two researchers throughout the study, however, the holding of the calves during the blood sampling was performed by students and hence varied throughout the study, which may have to some extent affected the stress levels the calves experienced.

Body weights were recorded at birth by the farmer, weekly on the dairy farm, upon arrival at the veal farm and carcass weights were recorded at slaughter. Calves were scored for clinical health every week at the dairy farm and in weeks 2, 6, 18 and 24 after arrival at the veal farm, using a standardised veterinarian protocol (Appendix 1). Rectal temperature was measured (°C) to assess fever (defined as ≥ 39.5 °C; Gomez et al.^45^). Navel, joint, fecal consistency, coughing, eye discharge, ear droop, nasal discharge and skin elasticity (dehydration) were scored: 0 (normal), 1 (slight, semi), 2 (severe), and the presence of sunken eyes was noted. In addition, individual treatments with antibiotics and other medicines (e.g. anti-inflammatories, anti-coccidiosis, vitamins) at the dairy and veal farm were recorded by the farmer.

Levels of immunoglobulins IgG, IgM, IgA were measured in the first milking colostrum, and four times in the serum of calves: in the first week after birth, the day prior to transport, and in weeks 2 and 10 after arrival of the calves at the veal farm^44^. See Marcato et al.^44^ for a full description of this analysis. In brief, colostrum samples of 15 mL were collected by the dairy farmer and stored in a freezer at −20 °C on the dairy farm. Thereafter, these samples were collected, processed (dethawed and pipetted 1 mL into micronic blocks) and stored at −80 °C until analysis. Blood samples of 10 mL were collected from the jugular vein of calves into serum vacutainer tubes (Vacuette, Greiner BioOne, Kremsmunster, Austria). The calves were first shaved around the sampling area. One person held the calf while another person blood sampled it. Blood samples were kept at room temperature until centrifugation (3000×*g* for 15 min at 4 °C). Serum was then decanted and stored at −20 °C until analysis. The titres of IgG, IgM, IgA in colostrum and serum samples were measured, using indirect enzyme-linked immunosorbent assay (ELISA) specific for phosphorylcholine conjugated to bovine serum albumin (PC-BSA).

The haematological profile of calves was assessed. Blood samples of 5 mL were collected from the jugular vein of calves into EDTA vacutainer tubes (Vacuette, Greiner BioOne, Kremsmunster, Austria) one day prior to transport and after two weeks at the veal farm. Samples were stored at 4 °C and then analysed by fluorescence flow cytometry (XT1800i, Sysmex Europe GmbH, Germany) for a complete haematological profile, including: haemoglobin (Hb), haematocrit (Ht), red blood cells (RBC), mean corpuscular haemoglobin (MCH), mean corpuscular volume (MCV), mean corpuscular haemoglobin concentration (MCHC), red cell distribution width (RDW), red blood cells (RBC), white blood cells (WBC), lymphocytes, neutrophils, monocytes, basophils and eosinophils^44^. See Marcato et al.^44^ for a complete description.

**Figure 2:**
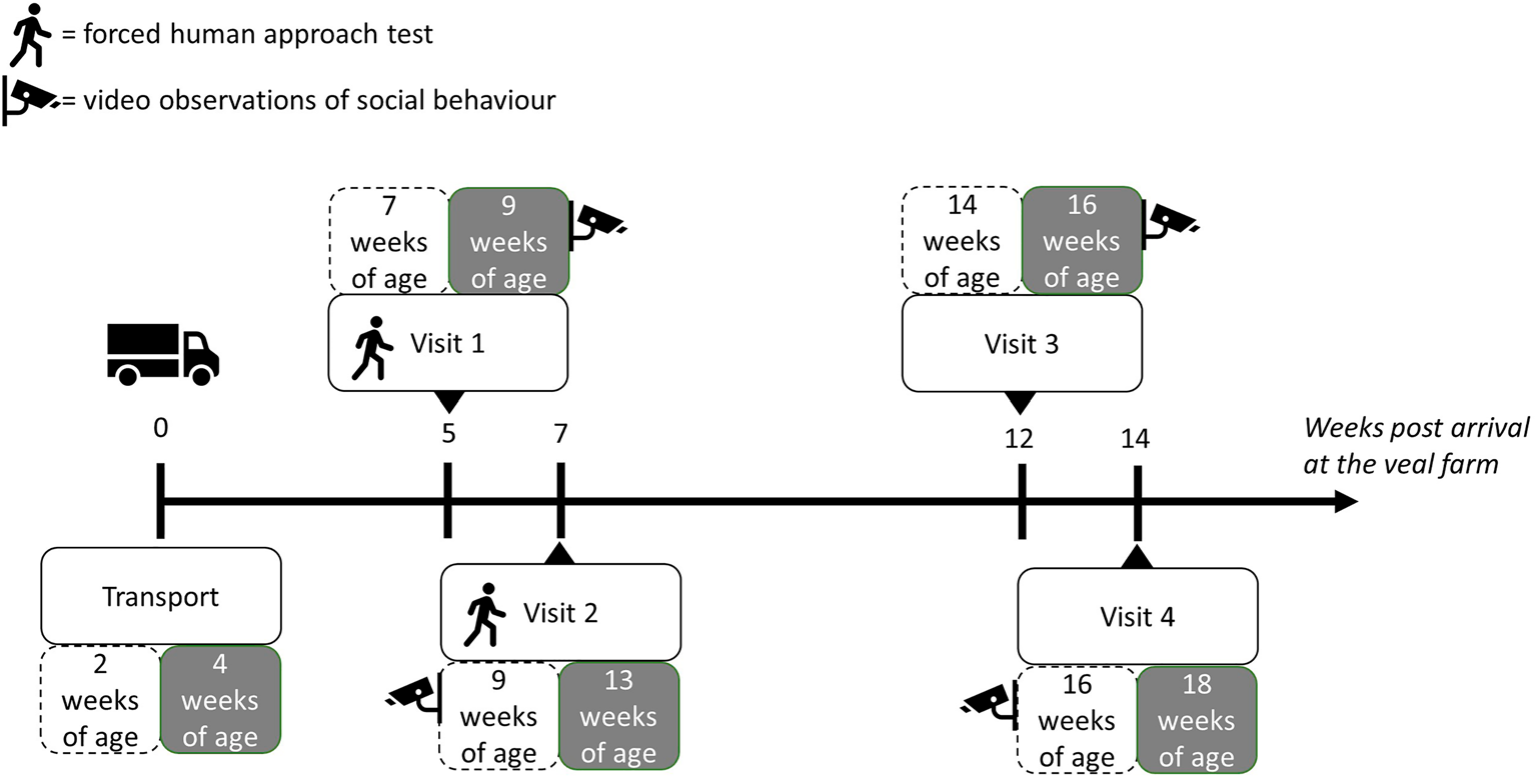
Schematic explanation of timing for forced human approach test, performed at 5 and 7 weeks after arrival at the veal farm, regardless of transport age, and video observations performed at 9 and 16 weeks of age, hence corrected for transport age. The dashed boxes represent the calves that were transported at 2 weeks of age, while the grey boxes represent the calves transported at 4 weeks of age.

The behaviour of the calves was recorded at the veal farms, pens being observed in a random order between milk replacer feeding times (approximately 6:00 and 16:00). A forced human approach test was conducted after 5 and 7 weeks at the veal farm (Fig. 2), to assess the calves' fear of humans. This test was done according to the Welfare Quality Protocol (Welfare Quality assessment protocol for cattle, page 127). The observer (the same throughout the entire study) entered the pen and stood in a corner immobile for 1 min. Then the observer tried to make eye contact with a hand extended forward, making sure the distance to the calf was approximately 2 steps (2 m). If eye contact was possible, the observer approached the calf with 1 slow step, then 1 s pause followed by another step. If the calf had not moved, the observer attempted to touch the head of the calf. The following scores were given: 0 = no eye contact was possible; 1 = eye contact, first step did not succeed, i.e. calf moved a leg; 2 = first step succeeded, second step did not; 3 = second step succeeded, calf cannot be touched; 4 = calf could be touched.

We investigated the effect of rearing, transport age and age of observation on various categories of social behaviour in calves, as well as on standing idle and oral contact with the environment which could indicate poor welfare in calves^46,47^. Behaviour was recorded via video recordings of the calves at the veal farms at 9 and 16 weeks of age (Fig. 2). Cameras were placed for 1 h in front of each pen. The time of day differed between pens, due to the availability of only 6 cameras, hence ability to film only 6 pens at once. At 9 weeks of age, the forced human approach test was carried out (Fig. 2), at the end of which the focal calf was sprayed with pink paint for identification in the videos, and the recordings of behaviour started 5 min after the observer had stepped out of the pen. At 16 weeks, there was no forced human approach test (Fig. 2) and the recordings started 5 min after the human left the pen following spraying of the focal calf. On some occasions, the focal calf could be sprayed from the feeding gate, hence without the observer entering the pen. However, in all cases, the observer ensured that all calves were standing prior to the start of the video to standardise the pens, which sometimes required the observer to enter the pen (it was never necessary to touch any of the calves for them to stand, simply approaching them was sufficient). Observations of these videos were done for 30 min (starting 5 min after the experimenter exited [at 9 weeks] or moved away [at 16 weeks] from the pen), using The Observer XT and a single observer. The ethogram used for the observations can be found in Table 1.

**Table 1:** Behaviours and definition used in this study. Categories used for analysis are provided^1^.

Category^1^	Behaviour	Description	Type
Standing idle		The focal calf is standing while not performing, or receiving any of the behaviours described below. It may, or may not, be moving its head while looking around, or it may be standing vigilant (i.e. with head raised and ears erect)	State
Contact environment		The focal calf’s muzzle is in close contact with any part of the environment, e.g. floor, barriers, trough. This includes, when observable, any chewing, licking or biting of the environment	State
Invisible		It is unclear what the focal calf is doing because the calf is not entirely visible	State
Other		Any state behaviour not described above (e.g. coughing, rubbing etc.). This behaviour fills the gaps between the other state behaviours and may run in parallel with other ‘event’ behaviours	State
Play*	Individual locomotory play	Jump, gallop, buck, buck-kick, fast turns, sometimes accompanied by headshake. Performed alone, i.e. no other calf in the pen or visible adjacent pens performs any of these elements	Event
Play + social (given^2^)	Parallel locomotory play	Jump, gallop, buck, buck-kick, fast turns, sometimes accompanied by headshake. The focal calf’s play is simultaneous with the play behaviour of other calves in the pen or adjacent pens (visible on the video)	Event
Play + social (given)	Frontal pushing	The focal calf is placed front to front to another calf in the pen while the two are mutually butting their heads against the head or neck of the other without this behaviour resulting in withdrawal	State
Play + social (given)	Mounting	The focal calf lifts both forelegs and then rests forelegs and sternum on the back, side or head of another calf	Event
Social (given)	Contact other calf	The focal calf’s muzzle is in contact with any part of the head or body of another calf in the pen or a calf from an adjoining pen	State
Social (given, negative)	Body pushing	The focal calf is pushing its head with force on any body part, except the head or neck, of another calf	State
Social (given, negative)	Disturbs lying calf	The focal calf is stepping on, hitting or otherwise causing other calves to stand up due to its activity	Event
Social (given, negative)	Displaces other calf	The focal calf body-pushes, or rests its head on the back of another calf and this causes the other calf to move (all four legs move) away (from trough, water, or position)	Event
Social (received)	Is contacted by other calf	A calf (from the same or another pen) has its muzzle in close contact with any part of the body or head of the focal calf. When the other calf walks away and then returns contacting the focal calf, this interrupts the behaviour and the behaviour is scored again	Event
Social (received, negative)	Disrupted lying	The focal calf is being stepped on, hit or otherwise made to stand up due to the activity of another calf	Event
Social (received, negative)	Is displaced	The focal calf moves (all four legs move) away (from trough, water, or position) due to the behaviour of another calf (e.g. head butting of the body, or rests its head on back of the focal calf)	Event
Social (received, negative)	Is being mounted	The focal calf is being jumped onto. The other calf lifts both forelegs and then rests its forelegs and sternum on the back, side or head of the focal calf	Event

*Jensen et al.^57^.

^1^State (durations) and event (counts) social behaviours were grouped into separate categories, which means that the following (categories of) behaviours were ultimately analysed: standing idle (duration), contact environment (duration), play (count), social interactions (duration), social interaction (count), social interaction given (count), social interaction received (count), negative social interaction (count).

^2^The terms given and received indicate whether the calf was the actor in the behaviour or was receiving the behaviour from another calf, respectively.

Milk yield parameters of the dams, including machine milk yield per day (kg/d), dry period length (days), and lactation length (days) were recorded automatically via four Lely A4 milking robots.

### Statistical analysis

Raw means and standard errors to the mean (SEM) are reported throughout, except for binary data for which percentages are provided. All statistical analyses were carried out in SAS© 9.4 TS Level 1M5 (SAS Institute Inc., Cary, NC, USA). The residuals of continuous data were checked for normality and homogeneity of variance. Data that met these two assumptions were analysed with a linear (mixed) models (L(M)M). Count data that did not meet these two assumptions were analysed with generalised linear (mixed) models (GL(M)M), specifying a Poisson distribution and log link function. Proportions were analysed with a GL(M)M specifying a binomial distribution and logit link function. All GL(M)M specified an overdispersion parameter. Mixed models specified the random effect of individuals to account for the dependency between repeated measures on calves or cows. Where relevant, random effects of veal farm were added (Table 2). In all analyses, approximate F-tests (Kenward and Roger, 1997) were used for fixed effects. Subsequent pairwise comparisons were done with Fisher’s LSD method. Response variables and analyses are described in Table 2. Further details are described in the section below per category of variables.

**Table 2:** Summary of the statistical analyses carried out in this study, including a description of response variables, time points and fixed effects. Abbreviations are described under the table.

	Variable	Type of variable	Time points	Time point analysis	Fixed effects^1^	Random effects
Body weight	Weight	Continuous normal	DF 1–5 weeks VF arrival Carcass	Separate	Rearing *Transport age* Breed(gender) Parity Assistance	*For time points at VF: random effect of VF added*
Health at dairy farm	Health	Binary	DF 1–5 weeks	Separate	Rearing Breed(gender) Parity Assistance	–
	Skin elasticity					
	Fever					
Health at veal farm	Health	Binary	VF 2, 6, 10, 18 weeks	Separate	Rearing *Transport age* Breed(gender) Parity Assistance	VF
Treatments (antibiotic AB and other medicine MED)	AB at DF	Binary	No repeat	Not applicable	Rearing Transport age Breed(gender) Parity Assistance	VF
	AB at VF					
	AB total					
	MED at DF					
	MED at VF					
	MED total					
Immunoglobulins*	IgA	Count	Colostrum (N = 37) DF 1 weeks DF pre-trans VF 2, 10 weeks	Separate	*Rearing* *Transport age* Breed(gender) Parity Assistance	*For time points pre-trans, VF2 and VF10: random effect of veal farm added*
	IgM					
	IgG					
Haematology	WBC, RBC, Hb, MVC, MCH, MCHC	Continuous normal	DF pre-trans VF 2 weeks	Together	Rearing Transport age Breed(gender) Time Parity Assistance	Calf
	Counts of: Lymphocytes Monocytes Neutrophils Eosinophils Basophils	Count				
	Percentages of: Ht Lymphocytes Monocytes Neutrophils Eosinophils Basophils	Proportion				
Fear of humans	Forced human approach score	Proportion	VF 5, 7 weeks	Together	Rearing Transport age Breed(gender) Time	VF(calf)
Social behaviour	See ethogram in Table 1	Proportion Count	Age 9, 16 weeks (VF)	Together	Rearing Transport age Breed(gender) Time No. calves in pen	VF(calf)
Dam performance	Dry period length	Count	No repeat	Not applicable	Separation Breed Parity Assistance	–
	Lactation length	Count	No repeat	Not applicable	Separation Breed Parity Assistance	–
	Milk production	Continuous normal	Lactation Weeks 1 to 6 Months 1 to 10	Together	Separation Breed Parity Assistance Time	Cow

DF = Dairy farm; VF = Veal farm; Ig = immunoglobulin; WBC = white blood cells; RBC = red blood cells; Hb = haemoglobin; Ht = haematocrit; MVC = mean corpuscular volume; MCH = mean corpuscular haemoglobin; MCHC = mean corpuscular haemoglobin concentration. *Fixed effects introduced into the model for Ig’s depend on the timepoint, see text.

^1^Fixed effects in italic are not included in all analyses at all time points. ‘Rearing’ and ‘transport age’ only applies to calves, not cows, and indicates whether they were reared with their dam or not, and at which age they were transported to the veal farm. ‘Separation’ treatment applies only to cows, not calves, and indicates the time from birth at which they were separated from their calf: 0, 2 or 4 weeks after birth. Breed is divided into two categories: meat breed with at least 50% Belgian blue and all other, termed dairy breed. Gender is nested in breed, because female calves were all of the meat breed. Parity is divided into three categories: 1–2, 3–4 and > 5. Assistance refers to assistance received during calving (yes or no). For social behaviours, the total number of calves in the pen was added as a fixed effect.

Initially, the interaction rearing treatment x transport age was included in all models where this was relevant (i.e. not on dairy farm data where the transport treatment had not yet been applied) and if found to be non-significant (P > 0.05), it was subsequently removed from the model and not further reported in the results section. The fixed effect of transport age was kept in the model, but is not described in the results for weight, health, immunoglobulin and haematology data, because this treatment is described in detail in two previous companion papers^43,44^.

Note that on most veal farms there were some pens with two experimental calves. The number of calves with at least one other experimental calf in the same pen were as follows: 2 calves on veal farm 1, 2 calves on veal farm 2, 8 calves on veal farm 5, 8 calves on veal farm 6, 6 calves on veal farm 7 and 9 calves on veal farm 8 (60 independent pens left in total, instead of 77). Calves were not independent from other calves in the same pen, calves in adjacent pens and in the same barn. However, accounting for pen and barn as random effects in our models meant that the models would not converge due to too few degrees of freedom and we hence decided to omit these factors.

Breeds of calves were divided into two categories: dairy breeds and cross-breeds with so called meat breeds, mainly Belgian Blue (BB). All female calves (see Fig. 1) were of the meat breed, and hence gender was nested within breed in all statistical analyses. In the results section, it is reported as breed(gender). Cow parity was divided into three levels, based on the distribution of cows across the different parities: parities 1–2, 3–4 and > 5. Cow breed was divided into four levels based on the percentage of HF genes: HF < 50, HF50, HF75 and HF100. Assistance at birth was also introduced as a fixed effect where relevant and was a yes/no factor: if any assistance was provided during birth the cow received a ‘yes’ for this factor, otherwise the cow received a ‘no’. Assistance consisted of pulling by hand, using a calving jack, a breeched calf, or help provided by the veterinarian. No C-sections were ever required.

For measurements at the dairy farm, the fixed effect of transport age was omitted, unless the measurement was taken the day prior to transport. Birth weights were not consistently recorded by the dairy farmer and were hence omitted from the analysis. On the dairy farm, we distinguished three health response variables: skin elasticity, health score and rectal temperature. Skin elasticity was transformed into a binary measure with level 1 combining scores 1 and 2. The other health variables (navel, joint, faecal consistency, coughing, abnormal breathing, eye discharge, sunken eyes, ear droop, nasal discharge) were all combined into a binary response variable with 0 indicating no health issues and 1 any number of health issues on any of the health variables. Fever was a binary response variable with a score 1 for rectal temperatures ≥ 39.5, and a score 0 for all lower temperatures. At the veal farm, only health scores at 2 weeks after arrival could be analysed, as thereafter disease was too rare. Skin elasticity and rectal temperature were not recorded at the veal farms.

For the colostrum samples (n = 37, due to the farmer’s failure to collect more), fixed effects included only parity, breed(gender) and assistance at birth. Scores of the forced human approach test (at 5 and 7 weeks after arrival at the veal farm, Fig. 2) were transformed into proportions by dividing them by the highest possible score (i.e. 4). For the analysis of social behaviour at the veal farm (at 9 and 16 weeks of age), state behaviours were analysed as proportion of total time (30 min) that the calf was displaying them. Event behaviours were analysed as counts. The number of calves in the pen (ranging from 4 to 10) was included in the model to control for this but results on this factor are not reported (it was occasionally but not systematically significant).

For dam productivity, cows were divided across three treatments: separation from the calf at birth (n = 30), after 2 weeks (n = 18) or after 4 weeks (n = 9). For dry period length and lactation length, 5 cows were deleted from the dataset as they had not completed the lactation period. Machine milk yield data included some missing days where the robot had not registered the yield or the cow had not visited the milking robot. This led to the following recording to be abnormally high. To prevent these strange recordings, all these errors were removed from the data set. Machine milk yield per day per cow was thereafter first averaged across weeks for the first 6 weeks and thereafter averaged across month for the first 10 months.

## Results

Results related to the treatment of transport age (2 versus 4 weeks) have been reported for these calves and others from 13 dairy farms (in total 683 calves) in Marcato et al.^43^, where health and performance data are presented, and in Marcato et al.^44^, where immunoglobulin and haematological data are presented. This paper will hence not focus on this particular treatment in detail, presenting instead a brief summary of these results, except for behaviour and milk production parameters which are not discussed in the two articles mentioned above.

### Body weights

At the dairy farm, rearing treatment had an impact on body weight in weeks 3 (P = 0.025), 4 (P = 0.008), and 5 (P < 0.001) but not in weeks 1 (P = 0.955) or 2 (P = 0.483). Dam-reared calves were heavier than separated calves (week 3: 64.3 ± 1.8 vs 58.5 ± 1.5; week 4: 75.0 ± 2.5 vs 63.0 ± 2.1; week 5: 84.1 ± 2.3 vs 69.9 ± 1.8 kg). In all the weeks at the dairy farm, no effect of breed(gender), parity or calving assistance was found on calf body weight (P > 0.1). At arrival at the veal farm, dam-reared calves were heavier than separated calves (69.8 ± 2.2 vs 63.2 ± 1.8 kg; P = 0.010). No effects of breed(gender) (P = 0.162), parity (P = 0.717) or calving assistance (P = 0.918) were found. At slaughter, carcass weight was unaffected by rearing (P = 0.871), breed(gender) (P = 0.343), parity (P = 0.237) or calving assistance (P = 0.183).

### Clinical health

Prevalence for the indicators of clinical health collected at the dairy and veal farm and across time are displayed in Table 3, while individual treatments with antibiotics and other medicines are described in Table 4. At the dairy farm in week 2 (note: at the dairy farm week is equivalent to age of the calf but not all calves stayed the same number of weeks at the dairy farm), there tended to be more calves from dams in parities 3–4 with signs of disease than calves from dams in parities 5–8 (88% vs 63%, P = 0.088). At the dairy farm in week 3, more dairy-breed males (note: breed effects could only be tested within males, because females were all of the meat-breed) had signs of disease compared with meat-breed males (83% vs 56%, P = 0.010) and more dam-raised calves had a fever compared with separated calves (25% vs 5%, P = 0.005). At the dairy farm in week 5 (note: only 4-week transport calves), there tended to be more dam-raised calves with signs of disease than separated calves (58% vs 27%, P = 0.056). At the veal farm, no impact of rearing, transport age, breed(gender), parity, or calving assistance were found on the prevalence of signs of disease in week 2. In weeks 6, 10, 18, and 24 the number of calves with health issues was too low for the model to converge (see Table 3 for percentages). The statistical models for use of antibiotics and medicines did not converge.

**Table 3:** Percentage of calves with at least one sign of disease (all health scores are combined into a binary variable, as present/absent), showing signs of dehydration (based on skin elasticity test) or with a fever (≥ 39.5 °C) across weeks on the dairy and veal farms. Significant differences (P ≤ 0.05) are indicated with*.

	Sign of disease			Dehydrated		Feverish	
	Week	Separated	With dam	Separated	With dam	Separated	With dam
Dairy farm	1	77	73	15	22	31	30
	2	79	70	32	43	5	19
	3	56	64	13	19	3*	25*
	4	57	58	7	0	0	8
	5	27*	58*	0	0	0	0
Veal farm	2	48	57				
	6	21	8				
	10	21	22				
	18	15	11				
	24	7	17

**Table 4:** Percentage of calves that were treated individually with antibiotics or other medicines (anti-inflammatory, anti-coccidiosis or vitamins) based on rearing practice.

	Reared by dam	
	No	Yes
**Antibiotics**		
Dairy farm	5	0
Veal farm	36	33
Total	41	33
**Other medicines**		
Dairy farm	8	0
Veal farm	36	36
Total	41	36

### Immunoglobulins

**Figure 3:**
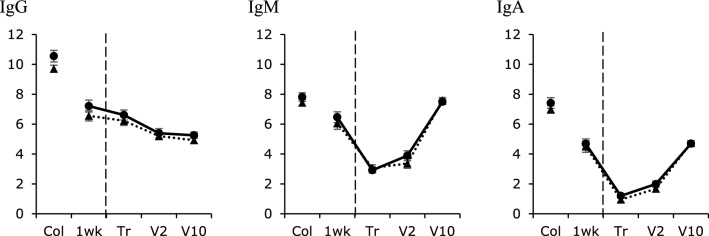
Immunoglobulin G, M and A titers (mean ± SEM) across rearing treatments (without dam = dotted line; with dam = continuous line). Levels are given across 5 collection time points based on analysis of dam colostrum and calf blood serum: Col = colostrum; 1 wk = at 1 week of age; Trp = the day prior to transport; V2 = after 2 weeks at the veal farm; V10 = after 10 weeks at the veal farm. The vertical dashed line indicates the start of the transport treatment. No differences between the rearing treatments were identified (P > 0.05).

The output of the analyses on immunoglobulins is shown in Fig. 3. Calves born with assistance had lower IgM titres (2.0 ± 0.2) than calves born without assistance (3.1 ± 0.1) prior to transport (P = 0.030). Rearing was not found to have an effect on IgG, IgM or IgA titers at any of the time points (Fig. 3).

### Haematological profile

The statistical output, as well as means and SEM for all haematological parameters across rearing treatment and sampling time is presented in Table 5. Regarding the other factors, males of the meat breeds had higher monocyte (P < 0.001) and basophil (P = 0.014) percentages than males of the dairy breeds. Within the meat breed, males had higher basophil percentage than females (P = 0.014). Calves from dams in parities 1–2 had higher counts (P = 0.002) and percentages (P = 0.008) of eosinophils than calves from dams in parities 3–4 and 5–8. Calves born with assistance had lower RBC (P = 0.049), Hb (P = 0.037) and Ht (P = 0.027) than calves born without assistance.

**Table 5:** Statistical output (raw means, P-values and SEM) for the haematological profile of calves reared with their dam or separated at birth, measured the day prior to transport and in week 2 at the veal farm. SEM is calculated across all data for that variable, regardless of treatment. Where the interaction between rearing practice and transport age was significant (P ≤ 0.05), the means are shown per transport age.

	Reared by dam			Sampling moment			SEM
	No	Yes	P	Prior to transport	Week 2 at veal farm	P	
WBC (10e9/L)	11.0	13.0	0.004	12.6	11.3	0.005	0.28
RBC (10e12/L)	8.8	9.2	0.128	8.5	9.3	< 0.001	0.11
Hb (mmol/L)	5.8	6.3	0.054	5.8	6.2	< 0.001	0.08
Ht (%)	27.2	29.2	0.054	27.5	28.6	0.003	3.34
MCV (fl)	31.1	32.0	0.275	32.6	30.7	< 0.001	0.23
MCH (amol)	661.4	684.9	0.076	689.0	660.0	< 0.001	4.23
MCHC (mmol/L)	21.3	21.4	0.174	21.2	21.5	0.001	0.08
RDW_SD	37.0	39.1	0.014	38.6	37.4	0.013	0.29
RDW_CV	33.4	34.0	0.470	33.2	34.1	< 0.001	0.19
Neutrophils (10e9/L)	3.5	4.1	0.974	4.9	2.8	< 0.001	0.22
Lymphocytes (10e9/L) 2 weeks transport 4 weeks transport	5.6 5.5 5.9a	6.5 5.9x 7.8b,y	< 0.001*	5.4	6.7	< 0.001	0.14
Monocytes (10e9/L) 2 weeks transport 4 weeks transport	16.1 17.3x 14.1a,y	19.6 18.8 21.0b	0.002*	19.1	16.4	< 0.001	0.50
Eosinophils (10e9/L)	0.13	0.20	0.367	0.25	0.07	< 0.001	0.02
Basophils (10e9/L) 2 weeks transport 4 weeks transport	0.13 0.14 0.11a	0.16 0.14x 0.20b,y	0.013*	0.13	0.15	0.020	0.01
Neutrophils%	29.9	30.2	0.448	37.5	23.0	< 0.001	1.10
Lymphocytes%	53.0	51.7	0.423	44.1	60.2	< 0.001	1.10
Monocytes%	14.8	15.3	0.910	15.3	14.7	0.166	0.30
Eosinophils%	1.2	1.5	0.407	2.0	0.7	< 0.001	1.76
Basophils%	1.2	1.3	0.237	1.1	1.4	< 0.001	0.05

WBC = white blood cells (10e9/l); RBC = red blood cells (10e12/l)); Hb = haemoglobin (mmol/l); Ht = haematocrit; MCV = mean corpuscular volume (fl);MCH = mean corpuscular haemoglobin (amol);MCHC = mean corpuscular haemoglobin concentration (mmol/l); RDW_SD = red blood cell width, standard deviation; RDW_CV = red blood cell width, coefficient of variance; *indicates where the interaction rearing treatment x transport age was significant (P ≤ 0.05): a-b indicates differences between rearing treatments while x–y indicates differences between transport ages.

### Forced human approach test

Scores on the forced human approach test at the veal farm were affected by rearing (P < 0.001) and visit (week 5 vs. 7; P = 0.011). Calves raised with their dam were more difficult to approach than calves separated at birth (average score: 1.3 ± 0.09 vs. 2.1 ± 0.13). Calves were easier to approach during the second visit than the first visit (week 5: 1.5 ± 0.10 vs. week 7: 1.9 ± 0.14). No effect of breed(gender) or transport age was found.

### Social behaviour at the veal farm

Results regarding social behaviour are displayed in Table 6. The duration of behaviours that have been linked to negative welfare in the past, i.e. standing idle and oral contact with the environment, did not differ between any of the treatments. However, the duration or frequency of all social behaviours, including ‘negative’ as well as given and received was higher in calves raised by their dam. Play behaviour could not be analysed due to too many zeros in the dataset. In addition, more social behaviours, albeit not negative social behaviours, were observed in calves transported at 2 weeks of age (Table 6). None of the behaviours investigated differed across the observation ages (9 vs. 16 weeks).

**Table 6:** Raw means, P-values and SEM for calf behaviour on the veal farm. See Table 1 for an explanation of which behaviours are included in each category below. SEM is calculated across all data for that variable, regardless of treatment.

	Reared by dam			Transport age			Observation age			SEM
	No	Yes	P	2 weeks	4 weeks	P	9 weeks	16 weeks	P	
**% duration of**										
Contact environment	13.8	12.0	0.910	13.1	12.6	0.236	12.1	14.1	0.286	0.9
Stand idle	10.9	12.2	0.228	11.5	11.7	0.705	12.4	10.4	0.145	1.0
Social interactions	7.4	9.2	0.063	9.5	5.5	0.084	8.7	7.7	0.143	0.8
**Count of**										
Social interactions	32.3	44.8	0.003	42.5	28.9	0.031	38.7	37.7	0.876	2.2
Social interactions given	21.2	27.2	0.021	26.7	18.1	0.092	24.1	24.8	0.999	1.5
Social interactions received	11.2	17.9	0.004	16.0	10.9	0.029	13.6	14.1	0.655	1.1
Negative social interactions	4.7	7.9	< 0.001	7.0	4.4	0.239	6.2	7.1	0.499	0.6
Play*	2.4	3.9	–	3.7	1.8	–	2.2	2.3	–	0.4

*Play model did not converge due to too many zeros in the dataset.

### Dam productivity

In the present study, body condition scores of the dams ranged from 3 to 5, with an average of 4.0 (SEM = 0.06) and did not differ between treatments. Dry period (after this experiment) and lactation lengths were on average 43.8 ± 1.8 and 342.8 ± 6.2 days, respectively. There were no effects of separation time (P = 0.117), breed (P = 0.687), or parity (P = 0.317) on dry period length of the dams, but there was an impact of calving assistance (P < 0.001): dams that received assistance during calving had on average a longer dry period length than dams that did not receive assistance (69.5 ± 14.7 vs 41.6 ± 1.2). Note however that there were only 5 dams that received assistance during calving in our study. There was no impact of separation time (P = 0.765), breed (P = 0.458), parity (P = 0.538) or calving assistance (P = 0.524) on lactation length.

**Figure 4:**
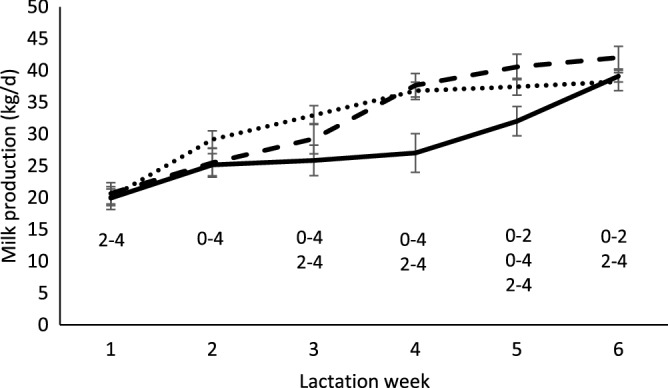
Machine milk yield (mean ± SEM) for the first 6 weeks of lactation of cows separated from their calves at birth (dotted line), after 2 weeks (dashed line) or after 4 weeks (continuous line). Significant differences between different separation times are indicated on the graph: e.g. 0–2 = 0 week and 2 weeks separation groups differ.

Looking at the first 6 weeks of lactation, there were effects of the separation x week interaction (P < 0.001; Fig. 4), breed (P = 0.005), and parity (P = 0.002) but not of calving assistance (P = 0.207). Cows with < 50% HF (23.0 ± 1.6 kg/d) had lower machine milk yields than cows with 50% (31.8 ± 0.8 kg/d), 75% (33.3 ± 1.0 kg/d) or 100% HF breed (33.4 ± 1.6 kg/d). Cows from parities 1–2 had lower machine milk yields (27.9 ± 0.9 kg/d) than cows in parities 3–4 (34.6 ± 0.8 kg/d) and parities > 5 (32.3 ± 1.1 kg/d).

**Figure 5:**
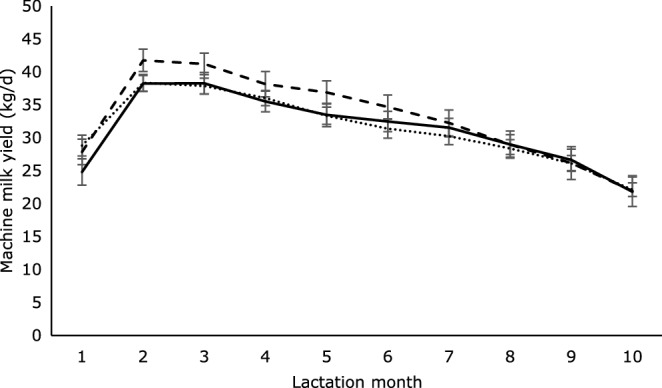
Machine milk yield (mean ± SEM) for the first 10 months of lactation of cows separated from their calves at birth (dotted line), after 2 weeks (dashed line) or after 4 weeks (continuous line). There was no effect of separation time and no interaction between separation time and month.

Machine milk yield per day, for 300 days of lactation, is displayed per treatment per month in Fig. 5. Looking at the first 10 months of lactation, there were effects of month (P < 0.001; Fig. 5), breed (P = 0.018), and parity (P = 0.001), but not separation (P = 0.115) or calving assistance (P = 0.178). Note that there was also no interaction of separation x month (P = 0.132), so this interaction effect was removed from the model. Cows with less than 50% HF genes had lower machine milk yield than cows with 50% or 75% HF genes (HF < 50 = 24.1 ± 1.0; HF50 = 32.6 ± 0.5; HF75 = 32.8 ± 0.7; HF100 = 31.3 ± 1.4 kg/d). Cows in parities 1–2 had lower machine milk yield than cows in parities 3–4 and cows in parities > 5 (1–2 = 27.8 ± 0.6; 3–4 = 34.4 ± 0.6; > 5 = 33.2 ± 0.7 kg/d).

## Discussion

The overall aim of this study was to investigate the impact of dam-rearing and its interaction with transport age on veal calf welfare and dam productivity.

### Weight, health, and immunity of calves

We expected dam-reared calves to have a combined advantage in terms of body weight, health and immune parameters, whereby we would see heavier calves with higher levels of immunoglobulins and white blood cells which would lead to better health overall. These expectations were, however, only partially confirmed. Dam-reared calves were indeed heavier than separated calves from week 3 onwards at the dairy farm and at arrival at the veal farm. A previous review reported that weight gains can be up to three times faster in dam-reared calves compared with separated calves in the first two weeks^18^. This weight advantage was in our study, however, lost by the time the calves were slaughtered. Previous research is inconsistent in terms of the effects of dam-rearing on weight gain following separation from the dam. In many studies the stress of separation and reduction in milk intake seem to cause a weight check in dam-reared calves around separation, but despite this, in previous studies there is a maintenance of the weight advantage acquired during suckling in dam-reared dairy calves post separation^14^. This discrepancy is likely due to the present study having shorter periods of dam-contact and higher stress levels resulting from the combination of abrupt separation, transport to the veal farm, mixing with unfamiliar calves, a high disease prevalence after arrival at the veal farm and a new feeding management and environment at the veal farm. In the present study calves were separated at an age at which they were still very much reliant on milk. Although they received milk replacer at the veal farm, the amount was likely lower than what they were drinking from the dam, the type of milk was different and they had to learn to drink from an open bucket or trough. These differences lead us to infer that the milk intake after arrival at the veal farm was likely lower initially in dam-reared calves, though this was not monitored. If calves are separated at an age at which milk is of lesser importance and calves are offered the opportunity to compensate by increasing their solid feed intake, then separation is likely to have less of an impact on the weight of the calves.

Moreover, this advantage in weight did not go hand in hand with a considerable difference in immunity, as calves had similar levels of IgG, IgM and IgA throughout the study regardless of rearing treatment. Dam-reared calves, however, did have higher WBC, and in the 4 week-transport group, dam-reared calves had higher counts of lymphocytes, monocytes and basophils (both prior to transport and after 2 weeks at the veal farm) compared with separated calves. The latter differences disappeared when proportions were considered. Higher counts of white blood cells could also mean dam-reared calves are fighting more infections/pathogens, which is in line with them having slightly worse health scores at the dairy farm. More dam-reared calves showed clinical signs of disease in week 5 at the dairy farm and a fever (≥ 39.5 °C) in weeks 2 and 3 at the dairy farm. Worse health combined with higher white blood cell levels has been previously identified in full contact dam-reared calves^48^. In the latter study and another, it was suggested that particular care should be given to the housing conditions of the calves with their dams to maximise hygiene and minimise the risk of morbidity^49^.

Dam-raised calves did tend to show higher levels of Hb than separated calves (both the day prior to transport and after 2 weeks at the veal farm), but no differences on any other parameters linked to anaemia, which limits conclusions here.

The original expectations were based on the following empirical evidence: although there is no agreement in literature in terms of the impact of dam rearing on respiratory health or Johne’s disease, there does seem to be a potential benefit in terms of diarrhoea^50^. Moreover, keeping calves with their dam seems to have a benefit on IgG absorption from the colostrum, independently of the amount of colostrum that is fed^29^. Advantages in health and IgG absorption are likely to translate to better growth. In addition, previous research found that calves kept with their dam were heavier, mainly because of an increased milk intake compared with control/separated calves^14^. Higher body weights at arrival at the veal farm have been linked to reduced risk for both morbidity and mortality, and a better ADG at the veal farm^33,36,51,52^. In the present study, these advantages were not detected, but this could be because decreased risk of morbidity is already detected from approximately > 50 kg body weight^33,36^, and our calves were on average ≥ 59 kg regardless of rearing treatment or transport age.

We expected calves transported at 4 weeks to have a combined advantage in terms of weight, health and immune parameters compared to calves transported at 2 weeks, whereby we would see heavier calves with higher levels of immunoglobulins which would lead to better health overall. These findings are not discussed in detail here as they can be found in the two sister papers with a much larger sample size of calves and dairy farms^43,44^. In brief, in this study 4 week-transport calves were heavier at arrival at the veal farm and at slaughter, did not display better health scores, and showed higher levels of lymphocytes and lower levels of neutrophils prior to transport to the veal farm and 2 weeks after arrival at the veal farm.

### Behaviour of calves

We expected calves reared with their dam to show more fear towards humans but to engage in more social behaviour with peers. These expectations were confirmed by the present study. Scores on the forced human approach test were lower for dam-reared calves than separated calves indicating that these calves were more difficult to approach. The averages indicate that dam-reared calves could not be approach at all and would move when the first step towards them was made, while separated calves could be approached by one step and would move away when the second step was made (approximately 1 m difference). Looking at individual scores 11 separated calves could be touched while only 3 of the dam-reared calves could be touched. This has practical implications for the welfare of these calves when handled by humans.

In first place, this could be explained by the lower contact with humans these calves received at the dairy farm. In second place, this result might be due to the handling of calves at the dairy farm being possibly somewhat negative in nature: once per week, the dam-reared calves were caught to be placed in a common pen where they were clinically scored and/or blood-sampled. The clinical health scoring and blood sampling continued on the veal farm, albeit not weekly. The dam-reared calves, hence, seldom received positive human contact and seldom received any contact at all, which easily explains why these calves were less approachable at the veal farm. The separated calves were fed by humans and hence received more contact and of a more ‘positive’ nature, making them likely more willing to interact with humans^16^. In third place, previous research suggests that the presence of the dam and socialisation of calves with her makes calves slightly less open to socialise with humans and hence more difficult to handle^53^. All calves seemed to become more approachable between the visit at 5 and 7 weeks after arrival at the veal farm, either because they habituated to the test or because of increasing approachability with age, possibly because of their association between veal farmer and feed.

In addition, dam-reared calves displayed social interactions more frequently than separated calves. This was true for all categories of social behaviour included in this study—i.e. given, received and negative social interactions. Previous studies found that calves and heifers that had been nursed by a cow displayed more submissive behaviour^22,54^ towards more dominant individuals, initiated and received more agonistic interactions^55,56^, showed a broader range or more social behaviours towards other calves^18,57^ and performed more social and solitary play^19,56,58^. In addition, dam-reared calves and cows seemed to have higher dominance ranks^55,59^. Overall, these results suggest that dam-reared animals develop better, more adaptable social skills, as suggested by Buchli et al.^54^. However, certain studies also found no difference in social behaviours^59,60^ or play^61^ between dam-reared and separated calves. Previous studies moreover found that dam-reared calves were more likely to approach novel foods^62^ and better at changing learnt behaviour^63^ pointing to an overall better ability in adapting to changing circumstances.

Interestingly, the calves transported at the younger age of 2 weeks also showed higher levels of social behaviours of all types except the ones referred to as negative in this study. Note that these calves were observed at the same age as the calves transported at 4 weeks, to cancel out the effects of age on behaviour and cognition, which means these differences cannot be explained simply by saying that younger calves are more social than older ones. If we use the frequency of social behaviour as a proxy for welfare in calves, then we might conclude here that transporting calves at a younger age is beneficial for their social development and welfare. The present data for play behaviour had many zero observations due to the short observation time. Play behaviour occurs sporadically and previous studies have included 24 and 48 h of observation of undisturbed behaviour to take this into account^64^. Although in the present study, the occurrence of play behaviour could not be analysed, numerically the play frequencies were higher in 2-week transport calves and dam-reared calves. Play, similarly to social behaviour, is also often used as a proxy for better animal welfare^64^, although this has been recently nuanced^65^. Of note, none of the proxies for ‘negative’ welfare, i.e. standing idle^47^ and oral contact with the environment^66^, were found to differ between rearing treatments or transport ages. These mixed results lead to us to emphasise the care that must be taken when drawing conclusions with such measurements of animal welfare: many measurements may not unequivocally reflect higher or lower levels of welfare when considered alone.

### Dam productivity

We expected that cows with their calves would have lower machine milk yield, but we expected this to rapidly increase back to the level of cows separated at birth, as soon as the calves were removed, and that there would further be no impact on dry period length and lactation length. This expectation was based on previous literature demonstrating that once calves are removed, the machine milk yield increases back to normal^14^. Note that the latter is still true if we only select the studies with Holstein, Friesian or Holstein–Friesian breeds published after 2000 from the aforementioned review article.

Keeping calves with their dams for 2 or 4 weeks did not seem to impact dry period length or lactation length of the dams. Machine milk yield was affected by genetic makeup and parity, with cows with less HF genes and cows in parity group 1–2, having a lower machine milk yield. Cows reared with their calf had a lower machine milk yield, but once the calf was removed, their yield increased back to that of cows separated at birth within two weeks. Interestingly, although the cows reared with their calf for 2 weeks seemed to have higher machine milk yield in the 10 months of lactation (based on means comparison), we found no significant differences between the different rearing groups. Previous studies, which implemented a dam-rearing period of between 42 (6 weeks) to 180 days (26 weeks) did not seem to point to long-term effects of dam-rearing on milk yield^14^, which is in line with our study.

### General discussion

This study only included one dairy farm, with a particular management and housing system and studies including a range of dairy farms are needed to assess the generalisation of these results to other systems. Separation of cow and calf at a later age causes distress and is one of the reasons for not applying dam-rearing on dairy farms. Due to surplus calves remaining only a short period of time at the dairy farm, gradual separation, which is found to decrease this distress, was not feasible in the present study leading to calves being abruptly separated and directly transported to the veal farm. A personal observation (L.E. Webb) is that due to cows calving in a group pen, mis-mothering occurred, whereby mothers of separated calves nursed other calves than their own. The dairy farmer further reported calves drinking from other cows than their own mother on several occasions. Mis-mothering likely makes it more difficult for the dam to bond with and care for her calf and there could be a risk of failed transfer of passive immunity or a risk the calf struggles to learn to drink from the udder. One interesting additional observation was the increase in (rejected) visits to the milking robot of cows subsequently separated from their calves (at 2 or 4 weeks post-partum) (personal observation by the farmer, data not available). Further studies might investigate this novel variable in the context of dam-calf contact systems, which may be linked to the ‘searching’ for the calf. In this study, we were not able to record the possible behavioural response of cows and calves to this abrupt separation, but previous studies suggest this would have been a stressful event for both^39^.

Another point for future investigation and thought is the stress experienced by the calves when they move between the dairy and veal farm. These calves are typically exposed to several stressors including two transport events between the dairy farm and veal farm (only one transport moment in the present study) interrupted by sorting at an assembly centre, the mixing with unfamiliar calves, a high pathogen exposure and subsequent disease prevalence and finally the stress of adapting to an entirely new environment and feeding regime^51,67,68^. Some of these stressors require careful consideration as to whether they could be mitigated or removed altogether. This would further help improve the robustness and welfare of these calves. In the present study, all calves were transported directly from the dairy farm to the veal farm which likely led to reduced exposure to stressors in our calves.

## Conclusion

The present study identified potential benefits of rearing veal calves with their dam for 4 weeks, including higher body weights (lost by the time of slaughter) and higher counts of white blood cells, as well as more frequent social behaviours at the veal farm. However, dam-rearing also incurred costs, including slightly more health issues and higher fear towards humans. Machine milk yield in the dams was only decreased when the calves were present, which means the economic loss due to loss of saleable milk may not be that large when calves stay with the dam for such a short period of time.

## Data Availability

Data and SAS scripts are available from corresponding author upon reasonable request.

## Appendix 1: Protocol for clinical observations

From dairy farm until week 3 at the veal farm:

- Navel score:
  - 0 = normal
  - 1 = slightly enlarged, not warm or painful, without discharge
  - 2 = Enlarged with heat, pain or discharge
- Joint score:
  - 0 = normal, no signs of joint problems
  - 1 = slight swelling, not warm or painful
  - 2 = swelling with pain, heat, slight lameness
- Fecal score:
  - 0 = normal
  - 1 = semi-formed, pasty
  - 2 = watery, shift through the bedding
- Cough score:
  - 0 = no cough
  - 1 = induced single cough
  - 2 = repeated spontaneous cough
- Eye score:
  - 0 = normal
  - 1 = small amount of ocular discharge
  - 2 = moderate amount of bilateral ocular discharge
- Sunken eyes:
  - 0 = normal, bright eyes
  - 1 = eyes markedly recessed into the orbits
- Ear score:
  - 0 = normal
  - 1 = Slight unilateral droop
  - 2 = head tilt or bilateral droop
- Nose score:
  - 0 = normal serous discharge
  - 1 = Small amount of unilateral cloudy discharge
  - 2 = bilateral, cloudy or excessive discharge
- Dehydration score (skin elasticity):
  - 0 = skin tent returns to normal < 2 s
  - 1 = skin tent returns to normal in 2–4 s
  - 2 = skin tent returns to normal in > 8–10 s

At the veal farm, from week 5 until the end of the production cycle:

- Cough score (n. of calves) (This is the first step. We start with the cough score at the veal farm. The person who conducts clinical observations spends 1 min/pen to look at the experimental calves, to check if these animals show signs of coughing):
  - 0 = no cough
  - 1 = induced single cough
  - 2 = repeated spontaneous cough
- Abnormal breathing (n. of calves)
  - 0 = normal
  - 1 = abnormal breathing
- Nose score (n. of calves):
  - 0 = normal serous discharge
  - 1 = Small amount of unilateral cloudy discharge
  - 2 = bilateral, cloudy or excessive discharge
- Diarrhea (at pen level):
  - Yes
  - No
- Thick manure (at pen level):
  - Yes
  - No
- White manure (at pen level):
  - Yes
  - No
- Bloated calves (n. of calves):
  - 0 = normal
  - 1 = full on one side
  - 2 = full on both sides
- Joint score:
  - 0 = normal
  - 1 = slight swelling, not warm or painful
  - 2 = swelling with pain, heat and lameness
- Lame calves (n. calves)
- Bursa problems (n.calves):
  - 1 = fore legs
  - 2 = hind legs
  - 3 = both fore and hind legs
- Skin infection (n. of calves):
- Condition (15–30% behind) (n. calves) (To check calf condition, compare our experimental calves with the rest of the stable (and not just the other calves within the same pen))
- Condition (> 30% behind) (n. of calves)
- General condition:
  - 0 = normal
  - 1 = slightly impaired, depressed
